# Complete Genome Sequence of Ehrlichia mineirensis, a Novel Organism Closely Related to Ehrlichia canis with a New Host Association

**DOI:** 10.1128/genomeA.01450-14

**Published:** 2015-01-22

**Authors:** Alejandro Cabezas-Cruz, Erich Zweygarth, Marzena Broniszweska, Lygia M. F. Passos, Múcio Flávio Barbosa Ribeiro, Marina Manrique, Raquel Tobes, José de la Fuente

**Affiliations:** a Center for Infection and Immunity of Lille (CIIL), INSERM, CNRS UMR, Université de Lille U1019 8204, Institut Pasteur de Lille, Lille, France; b Comparative Tropical Medicine and Parasitology, Ludwig-Maximilians-Universität München, Munich, Germany; c Department of Veterinary Tropical Diseases, Faculty of Veterinary Science, University of Pretoria, Onderstepoort, South Africa; d Departamento de Medicina Veterinária Preventiva, Escola de Veterinária-UFMG, Belo Horizonte, Minas Gerais, Brazil; e Departamento de Parasitologia, ICB-UFMG, Belo Horizonte, Brazil; f Oh No Sequences! Research Group, Era7 Bioinformatics, Granada, Spain; g SaBio, Instituto de Investigación en Recursos Cinegéticos IREC, CSIC-UCLM-JCCM, Ronda de Toledo, Spain; h Department of Veterinary Pathobiology, Center for Veterinary Health Sciences, Oklahoma State University, Stillwater, Oklahoma, USA

## Abstract

We report here the complete genome sequencing of Ehrlichia mineirensis, an Ehrlichia organism that was isolated from the hemolymph of Rhipicephalus microplus–engorged females. E. mineirensis is the best characterized Ehrlichia isolate from a novel cattle-related clade closely related to the monocytotropic pathogen E. canis.

## GENOME ANNOUNCEMENT

Ehrlichia species are the etiological agents of emerging tick-borne human zoonoses that inflict serious and fatal infections in companion animals and livestock. Ehrlichia species are tick-borne Gram-negative alphaproteobacteria that belong to the family Anaplasmataceae. Five Ehrlichia species are recognized, three of which can cause human ehrlichiosis (E. canis, E. chaffeensis, and E. ewingii) (1, 2). The complete genome of E. chaffeensis, E. ruminantium, E. canis, and E. muris were previously reported (3–6).

E. mineirensis was isolated from the hemolymph of Rhipicephalus microplus–engorged females and was characterized as a new species within the Ehrlichia genus (7). The organism has been maintained in the laboratory by continuous passage in the IDE8 tick cell line, where the ultrastructure was characterized (8, 9). Recently, we reported evidence that E. mineirensis evolved from a highly variable clade of E. canis under adaptive diversifying selection (10). For genome sequencing, the bacteria were grown in IDE8 cells, purified by Percoll density-gradient centrifugation (11), and the total DNA was extracted using TRI Reagent (Sigma, St. Louis, MO, USA). A next-generation sequencing (NGS) library was made starting from 800 ng of DNA using the NEB Next kit (New England Biolabs, Ipswich, MA, USA). The final library had a mean insert size of 516 bp. The library was then titrated by qPCR, denatured, equilibrated, and diluted for sequencing in MiSeq (Illumina). A total of 7.5 million pass-filter quality reads, 2 × 150-bp in length, were generated, which showed an average quality score above Q30 in more than 95% of the bases. The *de novo* assembly was performed with all the reads using SPADES (12). Prior to the assembly the overlapping reads were merged with FLASH (13). The contigs were aligned with Mauve (14) to the E. canis Jake genome (GenBank accession no. CP000107.1), detecting the presence of some contigs from Ixodes ticks. After massive BLAST comparison, only those contigs with similarity to Anaplasmataceae sequences were selected for annotation resulting in 182 contigs and 1,414,066 bases with an *N*_50_ of 32,220.

The genome of E. mineirensis consists of 1,414,066 bp, with 30.36% G+C content. The origin of replication (*oriC*), predicted by similarity to the E. canis Jake oriC region defined in the DoriC database (http://tubic.tju.edu.cn/doric/info1.php?ac=ORI10030069), seems to be placed in contig ehr000001 in the intergenic space upstream from the divergent genes encoding for uroporphyrinogen decarboxylase (EC 4.1.1.37) and cytochrome *c* oxidase, subunit III (EC 1.9.3.1), and 3 genes downstream of the *DnaJ* gene.

The genome of E. mineirensis was annotated using the BG7 system (15, 16) and resulted in 944 genes, including protein-coding sequences (CDSs), RNA genes, and pseudogenes. Of them, 322 genes encode proteins with enzymatic activity (with defined EC code), 51 encode for membrane proteins, 55 are involved in DNA repair, and 144 are related with oxidoreduction processes.

The availability of the E. mineirensis genome will allow comparative analysis to E. canis and E. ruminantium in studying the evolution of host specificity of Ehrlichia spp.

### Nucleotide sequence accession numbers.

The E. mineirensis genome sequence has been deposited in GenBank under the accession numbers CDGH01000001 through CDGH01000187.
